# Pharmacists’ professional and service qualifications for the care of Trans, Travesti, and Gender-Diverse people in southern Brazil: a grounded theory study

**DOI:** 10.3389/fmed.2026.1715137

**Published:** 2026-02-18

**Authors:** Livia Maria de Souza Gonçalves, Beverley D. Glass, Luciano Soares

**Affiliations:** 1 Postgraduate Program in Pharmaceutical Services and Policies (PPGASFAR), Department of Pharmaceutical Sciences, Federal University of Santa Catarina, Florianópolis, Brazil; 2 College of Medicine and Dentistry, James Cook University, Townsville, QLD, Australia

**Keywords:** cultural competency, graduate pharmacy education, health services for transgender persons, sexual and gender minorities, transgender persons

## Abstract

**Background:**

Trans, Travesti, and Gender Diverse (TTGD) populations face barriers to healthcare, limited by the qualifications of health professionals; however, information on pharmacists’ qualifications to deliver this care is scarce in the literature.

**Objective:**

To examine pharmacists’ professional and service qualifications to provide care for TTGD populations within the Brazilian Unified Health System (SUS) in a southern Brazilian capital.

**Methods:**

A Grounded Theory study was conducted with ten pharmacists working in primary and secondary healthcare, within the SUS. Participants were recruited through theoretical sampling and included pharmacists employed in SUS services within the municipality, excluding pharmacy residency trainees. Semi-structured interviews were conducted in participants’ workplaces, audio-recorded, transcribed verbatim, and translated into English. Data were analyzed using open, axial, and selective coding with NVIVO^®^ 14. The study received ethical approval from the Federal University of Santa Catarina and followed the COREQ guidelines.

**Results:**

Pharmacists’ qualifications were limited by insufficient undergraduate, postgraduate, and continuing education on TTGD healthcare. Contextual and intervening factors included cisheteronormative norms, regulatory constraints, and incidental engagement with TTGD narratives. Central phenomena involved knowledge gaps, communication difficulties, limited multidisciplinary collaboration, and a product-focused approach. Strategies ranged from respecting social names and pronouns to providing superficial service and referring TTGD users to specialized clinics. Consequences included feelings of not being qualified, negative emotions, and centralization of TTGD care in specialized services. Contact with trans narratives, however, facilitated more respectful and effective care.

**Conclusion:**

Structural and educational reforms, professional training, curricular integration, engagement with TTGD narratives, multidisciplinary collaboration, and full implementation of the National Policy for Comprehensive Health Care for Lesbian, Gay, Bisexual, Travesti, and Trans Populations are critical to enhancing pharmacists’ capacity to provide inclusive, equitable, and humanized care within the Brazilian SUS.

## 1 Introduction

Trans, Travesti, and Gender Diverse (TTGD) populations face persistent barriers to healthcare, with one of the most critical being the lack of professional qualifications to provide care for them (1). Qualification is a multidimensional and socially constructed process that goes beyond technical training. It encompasses knowledge and skills developed through education and socialization, reflecting the potential to perform tasks in the workplace while linking education and work within broader social and power relations (2–6). The term “Trans” is used as an umbrella category encompassing diverse gender identities whose gender differs from that assigned at birth. Within this category, “Travesti” refers to a culturally specific transfeminine identity deeply rooted in Latin American social and political contexts, while “Gender Diverse” describes identities and expressions that diverge from binary norms (7, 8). Addressing these qualification gaps, therefore, requires situating professional qualifications within the Brazilian health system and its policy frameworks for TTGD care.

The Brazilian Unified Health System (SUS) is a publicly funded, universal health system organized across primary, secondary, and tertiary levels of care, with Primary Health Care (PHC) serving as the main entry point. In Brazil, specific public policies have been developed to address the health needs of TTGD populations (9). These include the “Transexualizing Process” (PTr), a SUS-funded program that organizes access to gender-affirming healthcare services and procedures through specialized services across different levels of care, and the National Policy for Comprehensive Health Care for Lesbian, Gay, Bisexual, Travesti, and Trans Populations (PNSILGBT). Among other strategies, the latter emphasizes the integration of gender identity and sexual orientation into professional training and continuing education. Despite these advances, implementation lacks uniformity, and professionals often lack the necessary training to provide inclusive and equitable care (9, 10).

Within this context, examining how health professionals are prepared to engage with and operationalize these policies is essential. Pharmacists, increasingly integrated into interdisciplinary teams, play a strategic role in ensuring access to healthcare for TTGD people (11, 12). Their contributions extend beyond medicine management to include therapeutic follow-up, sexual and mental health support, and preventive care such as HIV pre-exposure prophylaxis. Strengthening these roles requires not only supportive policies but also curricular and continuing education strategies that meaningfully incorporate TTGD health into pharmaceutical training (11–15).

Despite the strategic involvement of pharmacists in TTGD healthcare and the existence of national policies aimed at promoting equitable care within the Brazilian SUS, the scientific literature addressing pharmacist training specifically focused on TTGD healthcare remains scarce. A global scoping review on pharmaceutical education in LGBTQIA+ health did not identify Brazilian studies focusing on training or educational strategies for pharmacy students or pharmacists related to TTGD care (15).

Evidence from a recent scoping review on healthcare access for TTGD people in Latin America highlights the significant and complex role of pharmacies, particularly in relation to hormone use, as they often represent one of the most accessible points of care amid broader barriers to other health services. Nevertheless, the review indicates that pharmacists’ social, cultural, political, and educational backgrounds, as well as their practices related to TTGD users, remain underexamined compared with those of other health professionals (16). This combined lack of published evidence and structured educational initiatives constrains the understanding of how pharmacists are prepared to operationalize existing policies in everyday practice, and contextualizes the relevance of the present study, reinforcing the need to investigate pharmacists’ and their services’ qualifications as potential contributors to reducing inequities in healthcare access.

In light of this evidence, given the multiple pharmaceutical services across all levels of care in the SUS (17), and the professional’s relevance to TTGD healthcare (12), analyzing their qualifications to provide care for TTGD populations is essential to guide training, strengthen services, and address the literature gap on how pharmaceutical practice can advance healthcare equity. Therefore, this study aimed to analyze the pharmacists’ professional and service qualifications to provide care for TTGD populations within the SUS in a Brazilian capital.

## 2 Theoretical framework

Qualification is examined as this study’s central phenomenon. It is not restricted to individual skills or technical training but is understood as a socially constructed and historically situated process. From this perspective, work organization and professional training cannot be dissociated from the social relations, power structures, and institutional arrangements in which they are embedded. Qualification is therefore a multidimensional concept that encompasses the qualification of the job, the work process, and the worker, shaped by economic, social, and political contexts and by the technical and social regulations that govern professional practice (3–5). This conceptualization allows qualification to be examined beyond formal education, capturing how pharmacists’ roles, competencies, and practices are produced and negotiated in everyday work settings to provide care for TTGD healthcare users.

In this article, the term “healthcare users” refers to individuals who access and interact with health services. Although “patients” remains common in biomedical literature, we adopt “users” in line with critical public health perspectives that emphasize agency, autonomy, and rights beyond the traditional patient role (18, 19).

This study is theoretically informed by Blumer’s Symbolic Interactionism, which emphasizes that meanings are constructed through social interaction and continuously interpreted and modified by social actors. According to this perspective, individuals act based on the meanings they attribute to objects, situations, and relationships, and these meanings emerge from and are modified through interaction. In the context of healthcare, professional practices are understood as the result of continuous interpretive processes through which actors define situations, negotiate roles, and respond to institutional and social expectations (20).

The theoretical–analytical coherence of this study is ensured by the Grounded Theory method, described by Corbin and Strauss, an approach rooted in Symbolic Interactionism. Within this approach, social reality is viewed as dynamic and processual, constructed through actions and interactions (21, 22). The coherence between theory and method underpins an inductive analytical process guided by theoretical sensitivity and constant comparison. This integration enables the construction of a substantive theory that captures qualification as a dynamic and interactional phenomenon, produced and transformed through pharmacists’ everyday practices within the Brazilian Unified Health System.

## 3 Materials and methods

### 3.1 Study design

To develop a substantive theory, this qualitative study applied the Grounded Theory method developed by Corbin and Strauss (21, 22) to investigate the qualifications of pharmacists and pharmaceutical services to provide care for TTGD people within the Brazilian Unified Health System (SUS). In line with methodological standards, reports followed the COREQ guidelines (23).

### 3.2 Participants and recruitment

Eligible participants were pharmacists employed in the SUS in a southern Brazilian capital. Inclusion criteria included: (i) holding a valid pharmacist license; (ii) being formally employed in the SUS at the primary or secondary care level; and (iii) currently providing pharmaceutical services in this city. Although the municipality includes pharmacy residents who support service delivery as part of their professional training, they were excluded from the study because they are not contracted staff and remain under supervised training.

The theoretical sampling strategy proposed by Corbin and Strauss was used, whereby participant recruitment and data analysis occurred iteratively (21, 22). Initial participants were selected to explore broad aspects of pharmacists’ qualifications to provide care for TTGD people. As data analysis progressed, subsequent participants were recruited to further elaborate, refine, and contrast emerging categories and their properties.

Participant recruitment was supported by the city’s pharmaceutical services department, part of the local health authority, which distributed email invitations presenting the study and inviting pharmacists to participate. Additionally, potential participants were contacted by telephone at their workplaces by the research team.

A total of ten pharmacists participated in the study. Recruitment was concluded when theoretical saturation was achieved, that is, when no new properties or dimensions of the emerging categories were identified through continued data collection and analysis.

### 3.3 Data collection

Twelve in-depth one-on-one interviews were conducted from September through December 2022 until data saturation was achieved, defined as the point at which no new categories emerged from the analysis. Theoretical saturation was reached during the final stages of data collection and analysis (21, 22). Each interview lasted 22–50 min (average 36 min). The interviews were conducted in person in a private room at the participants’ workplaces, audio-recorded with permission, and complemented by field notes taken by the interviewer. Eight participants were interviewed once, and two participants were interviewed twice, as additional interviews were needed to address analytical gaps in the emerging theory until theoretical saturation was achieved.

Data were collected using a semi-structured interview guide designed to explore key dimensions of the qualification process, including service and work relationships, social and cultural processes, and professional training and practice. The guide consisted of five open-ended main questions and a demographic section aimed at characterizing participants’ socioeconomic profiles. Each main question was accompanied by suggested prompts and guidance on the type of content to be explored, which were used flexibly to deepen participants’ accounts when necessary (Table 1). Interviews typically began with a brief, informal conversation about service structure and organization to establish rapport before progressively addressing the study’s central topics.

**TABLE 1:** Interview guide question samples.

Category	Items
Main question	How is the service you work with organized?
Supporting questions	1. What services are offered at your workplace? 2. What professionals and technologies are involved? 3. What medicines are dispensed there? 3.1 What SUS pharmaceutical components are involved (basic, statetig, specialized)? 3.2 Are there medicines for hormonization? 4. Is there any service to follow up on users’ pharmacotherapy? 5. Has any activity in pharmaceutical care been implemented?
Explanation	The guidance for this question refers to their knowledge and perceptions about the workplace’s general activities, its scope, resources, and the professionals involved, regardless of the people it is intended for.

The interview guide was validated by three experts (two cis and one trans), who assessed its relevance and applicability to the phenomenon under investigation. A pilot interview was conducted with one pharmacist who expressed interest in participating in the study. The pilot aimed to prepare the interviewer and to refine the interview guide; following this process, minor adjustments were made to the instrument before commencing data collection with the remaining participants. All interviews were conducted by the first author, a cis woman and Ph.D. candidate.

The research team’s professional and academic background informed the study throughout all stages. The first author had experience as a pharmacist in SUS primary healthcare facilities and at the state pharmaceutical services coordination, had conducted previous research on TTGD healthcare, and participated in advocacy initiatives for LGBTQIAPN+ rights. The second author had experience researching pharmaceutical services for trans people in northern Australia, and the third author had extensive knowledge of Brazilian pharmaceutical services through academic teaching and research. Additionally, as pharmacists who are professors and Ph.D. students, they have studied inclusive and respectful pharmaceutical care through their professional careers. These combined experiences, together with formal training in grounded theory, guided study design, data collection, and analysis, supported continuous reflexivity and sensitivity throughout the research process.

### 3.4 Data analysis

The interviews were audio-recorded, transcribed verbatim, and carefully translated from Portuguese into English, with two researchers independently reviewing the transcripts to verify completeness and consistency, and translations reviewed by two professional translators to ensure accuracy and enable full team participation. The first author conducted data coding.

Analysis followed the three steps proposed by Corbin and Strauss: open, axial, and selective coding. The relational paradigm model was applied as a heuristic tool to organize relationships among emergent categories, including causality, context, phenomenon, intervening conditions, strategies, and outcomes, allowing theory to be generated inductively from the data (21, 22).

Open coding was the initial stage, during which transcribed interviews and field notes were read repeatedly and analyzed line by line, naming, comparing, and developing categories, using NVivo 14^®^ software. The data were separated into discrete parts, examined, and compared to identify recurring patterns. Concepts were labeled, grouped, and organized into preliminary categories that represented meaningful phenomenon (21, 22).

Axial coding is the process of relating categories to subcategories. It involved reassembling these fractured or separated data by exploring the relationships among categories and subcategories, particularly how they connect in terms of causal conditions, context, actions/interactions/strategies, and consequences. This phase also emphasized identifying social processes, especially participants’ actions in response to the described conditions. During this process, codes were refined, properties and dimensions of categories were described, and analytical memos were developed to support interpretation (21, 22).

Selective coding was the final stage, in which a central (core) category was identified and systematically integrated with other categories to build a coherent grounded theoretical explanation, validated through relationships and refined by further developing categories as needed. Relationships between categories were organized using a paradigm model encompassing causality, context, phenomenon, intervening conditions, strategies, and outcomes. Constant comparison was applied throughout to ensure analytical consistency and depth (21, 22). The achievement of data saturation was thoroughly discussed among the researchers while examining the properties of the categories.

The core category integrates key dimensions that characterize pharmacists’ current qualifications to provide care for TTGD healthcare users within pharmaceutical services. It was constructed by integrating categories that conceptually aligned with the analytical definition of professional and service qualification adopted in this study. Categories were incorporated into the core when they meaningfully represented dimensions of pharmacists’ and pharmaceutical services’ qualification to deliver care for TTGD people within the public healthcare context.

Relational hypotheses were subsequently developed through analytical statements to explain how the central phenomenon is influenced by causal, contextual, and intervening conditions, and how these conditions shape participants’ actions, interactions, strategies, and their consequences (21, 22). The paradigm model was used as a heuristic tool to support this integrative process, rather than as a rigid framework, enabling the construction of a grounded theoretical model that captures the dynamic and multidimensional nature of pharmaceutical services’ qualifications to provide care for TTGD people.

The relevance and centrality of categories were determined by their analytical development, observed variation across empirical instances, and explanatory contribution to the emerging theory. Through constant comparison, concepts were progressively abstracted from participants’ accounts and elevated to categories when they demonstrated conceptual relevance and sufficient variation. Relationships between categories were analytically examined, and centrality was attributed to those that consistently contributed to explaining the central phenomenon across different contexts and conditions (21, 22).

Although the occurrence of categories across interviews was monitored as an auxiliary indicator of analytical robustness, prioritization was guided by theoretical sensitivity, conceptual density, and explanatory coherence rather than by quantification. The designation of centrality, therefore, reflects the integrative role of categories within the grounded theoretical model, as refined through selective coding and theoretical saturation (21, 22).

Throughout the analysis, data and emerging categories were continuously compared, and interpretations were discussed, validated, and refined with two experienced pharmacy professors. Procedures, including duplicate transcript review, constant comparison, peer discussion, and careful transcription and translation, ensured trustworthiness while embedding the researchers’ preparation, reflexivity, and professional positioning within the study process.

### 3.5 Ethical consideration

This study was approved by the Human Research Ethics Committee of Santa Catarina Federal University (CAAE: 59891822.0.0000.0121). Participation in the study was subject to the signing of the Informed Consent Form by participants, which invited the participants to join the research, presented the study’s purpose and methods, explained the activities and the approximate duration of the interview, data management and storage, as well as the researcher’s commitment to confidentiality. This document also presented the research group, the risks and benefits of participating, and assured them that they could pause or end interviews at any time. This information was verbally emphasized before the beginning of the interview, when the interviewer explained that the interview would be recorded for transcription purposes, that data would be securely stored and discarded after the completion of the research, and that participants’ confidentiality would be maintained. No one refused to take part or withdrew from the study. Participants are referred to using the singular, gender-neutral pronouns they/them to protect anonymity.

## 4 Results

The characteristics of the pharmacists are summarized in Table 2. From the ten participants, eight identified as white, and two as *pardo* (as classified in the Brazilian census). Regarding their socioeconomic status, all participants were classified as middle class. Seven reported a household monthly income ranging from BRL 4,848.01 to BRL 12,120.00 (approximately USD 893 to USD 2,231), and three reported incomes ranging from BRL 12,120.01 to BRL 24,240.00 (approximately USD 2,231 to USD 4,462). The participants had academic backgrounds ranging from college graduates to those with graduate degrees, all of whom graduated with a pharmacy degree from public universities. Nine of them had a postgraduate course, 7 had one or more Professional and Continuing Education courses done in private and/or public institutions, 6 had a master’s degree undertaken in a public university, 2 started their Ph.D. but didn’t complete it, and one completed it in a public university. Time since pharmacy degree ranged from 5 to 40 years (median = 18.55 years). Time spent studying (undergraduate and postgraduate studies) varied from 3 to 20 years (mean: 10.85 years).

**TABLE 2:** General characteristics of participants.

*N*	Gender identity	Age (years)	Sexual orientation	Years spent studying	Years since pharmacy degree	Education level	Continuing education*
1	Cis man	39	Homosexual	20	17	Master’s	Yes
2	Cis woman	31	Heterosexual	14	7	Ph.D.	No
3	Cis woman	40	Heterosexual	10	5	Bachelor’s	Yes
4	Cis woman	43	Heterosexual	10	22	Master’s	Yes
5	Cis woman	48	Heterosexual	3	23	Bachelor’s	No
6	Cis woman	53	Heterosexual	8	28	Bachelor’s	Yes
7	Cis woman	38	Heterosexual	12	15	Master’s	No
8	Cis woman	47	Heterosexual	9	23	Master’s	Yes
9	Cis woman	62	Heterosexual	12	4	Master’s	Yes
10	Cis woman	29	Heterosexual	9	5	Bachelor’s	Yes

*Master’s and doctoral degrees were not included under “continuing education,” as these confer academic titles already listed in “education level.”

All participants worked in public community pharmacies within the Brazilian Unified Health System (SUS) in southern Brazil. Half were allocated to pharmacies located within primary healthcare centers, which operate as open-access services and are organized according to the Family Health Strategy model, with multiprofessional teams responsible for defined territories and longitudinal care. These units operate within the principle of comprehensiveness, coordinating care across different levels of the health system and serving as the main entry point for the local population.

The remaining participants worked in secondary care facilities: one centralized pharmacy dedicated to dispensing high-cost and specialized medicines, operating independently from other healthcare services and serving as the only municipal reference for access to these medicines; and another pharmacy linked to two services located within the same building, a general polyclinic and a trans-specific outpatient clinic. In the Brazilian SUS, polyclinics provide specialized outpatient care and are accessed through a referral and regulatory system, usually initiated in primary care.

The trans-specific outpatient clinic provides multidisciplinary care for Trans, Travesti, and Gender-Diverse people across the life course. Services include medical, nursing, and psychological care; support during gender identification processes; initiation and follow-up of hormonization; sexual and reproductive health care, including HIV and STI testing, prevention, and treatment (PrEP and PEP); gynecological preventive care; and guidance on legal and administrative processes related to civil registry rectification. The clinic also offers referrals and clinical reports required for gender-affirming procedures, operating as a specialized reference service within the local health system. The related pharmacy primarily dispensed medicines for the management of conditions of epidemiological relevance or affecting vulnerable populations, such as HIV/AIDS, and also provided access to medicines for hormonization. To ensure anonymity in the publication, participants’ names were replaced with colors, which will be used throughout the text to refer to each of them: Purple, Pink, Yellow, Green, Blue, Gray, Orange, Golden, Red, and White.

During the axial coding process, previously formed concepts that could be merged based on their properties formed larger categories and became the subcategories/concepts, whereas those with consistent constructs that did not merge emerged as unique single categories without further subdivisions. The data analysis of the Pharmacists’ Professional and Service qualifications to provide health care for TTGD people, through the categorization of the interview data coding processes, yielded 37 main categories and 45 subcategories (Table 3).

**TABLE 3:** Categories and subcategories of pharmacists’ professional and service qualification to provide healthcare for TTGD people in southern Brazil.

Category	Subcategory
Lack of academic experience related to healthcare for TTGD people	Received (or not) training on TTGD-healthcare-related issues
	Realize the lack of training on TTGD healthcare
Lack of workplace training on healthcare for TTGD people	Isolated experiences of on-the-job training in TTGD healthcare
	No on-the-job training on TTGD healthcare
Coloniality: the modern colonial CIStem of gender and capitalism	General precarious services due to Neoliberal policies and the dismantling of the healthcare network
	Cisnormative values as a social standard
	University’s mandatory subjects run according to CIStem’s norms – excluding TTGD people
	Healthcare services reproduce discrimination against TTGD people
	Exclusion of TTGD people in cisnormative networks
Healthcare regulation structuring specialized services and PHC referral-counter-referral systems	The presence of one outpatient clinic specialized in TTGD people’s healthcare in the city’s public network.
	PHC services are decentralized and responsible for acting as the center of communication between healthcare points
	Communication failures in the health services network
Poor PNSILGBT implementation in service	–
Changes in the community pharmacist profile/scope of practice	–
Social, cultural, and political environments and values	Influence of taboos and prejudices
	Political environmental discussions
	Contact with friends engaged in TTGD healthcare
Occasional experiences related to TTGD people in academic activities and work environments	Extracurricular academic experiences
	Discussions with the team
Relate with narratives of TTGD people	Through social movements
	Through the personal environment
	Through work experiences
Lack of pharmacists’ knowledge of TTGD people’s healthcare and general concepts	TTGD-related concepts
	TTGD-specialized services
	Related public policies
Difficulty in communicating with TTGD users	Feel embarrassed and uncomfortable when assisting TTGD people
	Don’t know what name to call the user
	Don’t know the user’s personal pronouns, but don’t ask them
Denaturalization of TTGD identities	Try to act naturally with TTGD users, despite not considering their identity “natural”
	Feel surprised when seeing a TTGD user
	Show taboos or prejudices against TTGD people
Limited pharmaceutical involvement in multidisciplinary teams	Work in multi-professional teams
	Intensity of pharmacist collaboration with other professionals
Pharmaceutical services focused on product delivery	Dispensing medicines (properly or delivering them)
	Managing administrative processes to access medicines
	Incipience of other healthcare initiatives
Greater contact by pharmacists linked to the TTGD outpatient clinic	Very often see TTGD people in the service
	Used to see TTGD people in the service often, but it has reduced
	Rarely seeing TTGD people in the service
Actions regarding the use of the user’s social name and personal pronoun	Asking for the social name pronoun when in doubt
Calling the user by the wrong (not the social) name
Interactions with peers toward adverse situations related to TTGD people’s healthcare	Point out colleagues’ mistakes
	Give training to colleagues
	Asking colleagues about their doubts
Providing fast and superficial care services to all users	–
Avoiding deeper care/discussion with TTGD users	–
Guiding Pharmaceutical services in a cisnormative way	–
Referring TTGD users to other services	–
(Un)preparedness feelings	Feel unprepared to provide care related to hormone use
	Feel unprepared to provide general care for TTGD people
	Feel prepared to provide care for TTGD people
Negative feelings when making mistakes related to the user’s name or gender identity	–
Feelings of being distant from the users/not having a link with them	–
Perception of the need for more training to provide care for TTGD people and willingness to learn more	–
Realize the need to insert TTGD health issues in pharmacy curricula	–
Superficial pharmaceutical care for TTGD people	–
Centralization of TTGD people’s healthcare in specialized services	–

**FIGURE 1:**
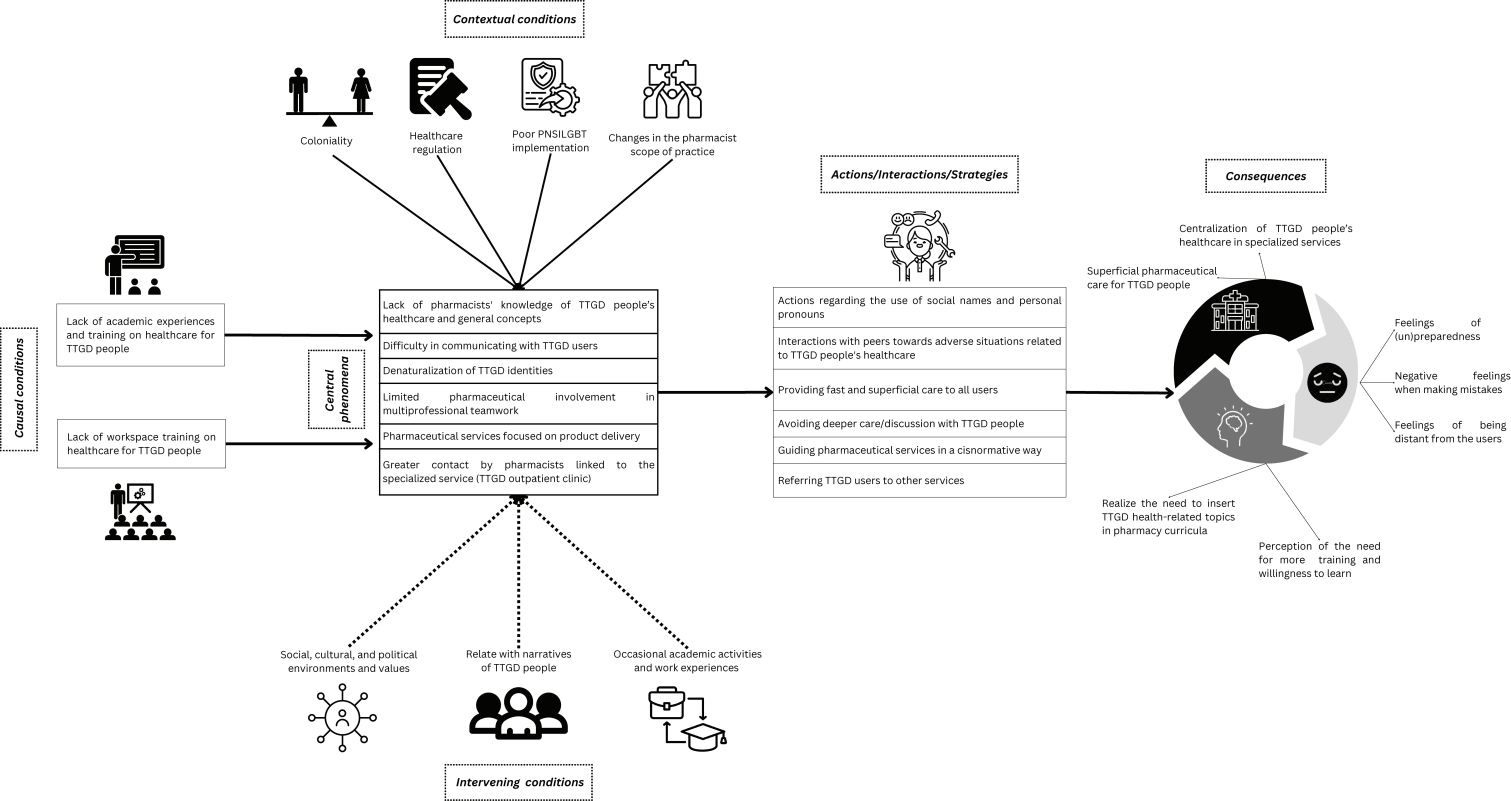
Grounded theory paradigm. Created with Canva.com.

Then, the categories were placed in the situation model according to their relationships, integrating: causal conditions (factors that give rise to the phenomenon), contextual conditions (broader situational or structural influences), intervening conditions (factors that directly affect participants’ responses to the phenomenon), the central phenomenon (the main process under study), actions/interactions/strategies (responses or behaviors related to the phenomenon), and consequences (outcomes resulting from the phenomenon and strategies) (21, 22). The organization of these categories and subcategories within the paradigm is visually represented in Figure 1, which illustrates the Grounded Theory model of Pharmacists’ Professional and Service qualifications to provide health care for TTGD people in southern Brazil.

The term “social name” is used in Latin America to refer to a person’s chosen name, equivalent to the “chosen” or “affirmed name” in Northern contexts, and will be consistently applied throughout this text (24).

### 4.1 Central phenomenon

The core categories represent the central social phenomenon of the study. These categories explain the phenomenon by answering the questions: “What is the main concept presented in this research? If my findings are to be conceptualized in a few sentences, what do I say? What does all the action/interaction seem to be about? How can I explain the variation that I see between and among the categories?” (21, 22).

In this study, the central phenomenon (pharmacists’ professional and service qualifications to provide healthcare for TTGD people) is described through six core categories, which are further divided into subcategories.

#### 4.1.1 Lack of pharmacists’ knowledge of TTGD people’s healthcare, and general concepts

This category arises from participants’ generally limited understanding of TTGD-related healthcare, despite differences linked to intervening conditions. It was developed from three subcategories: TTGD-related concepts, TTGD-specialized services, and relevant public policies. Most pharmacists acknowledged that their knowledge was insufficient to provide adequate care for TTGD users.

The majority of participants demonstrated uncertainty when explaining TTGD-related concepts, such as trans/transgender, cis/cisgender, and diverse gender identities (Travesti, trans woman, trans man, non-binary, among others). Several participants also showed confusion regarding broader LGBTQIAPN+ terminology, often conflating gender identity with sexual orientation, and, in some cases, with sex characteristics and intersex identities. Pink was able to distinguish between cis and trans identities, but their understanding of gender remained limited to a binary framework. Only Purple and White articulated TTGD-related concepts clearly and accurately. Furthermore, most participants reported being unaware of existing public policies related to TTGD health care.

Most pharmacists demonstrated limited knowledge regarding hormone use and were aware of these limitations. Half of the participants knew that the TTGD outpatient clinic is part of the public network and how it works. Two (Yellow and Pink) knew it existed, but didn’t know about the services provided there, and three (Golden, Gray, and Red) didn’t know about any TTGD-specific services provided in the public network in the municipality. The five participants who showed more knowledge had previously worked or were working in a pharmacy linked to the TTGD outpatient clinic. Even these missed some relevant information about the TTGD-outpatient services, such as White, who worked in a health center that used to be linked to the TTGD outpatient clinic, and wasn’t aware that medicines for hormonization were currently dispensed there, and Blue, who was working in the pharmacy linked to the TTGD outpatient clinic, but didn’t know about the clinic origin and management. In Latin America, the term “hormonization” refers to hormone use and affirms gender diversity as a human right, opposing biomedical pathologization (25).

- *“I remember our confusion was because of that, because there were two names [for the same user], you know? (…) He was a man, but trans woman, right? I don’t know how to say it. (…)” Golden*

#### 4.1.2 Difficulty in communicating with TTGD users

This category can be described as the participant’s practices that indicate difficulty in communicating with TTGD people and was created from the subcategories: “Feel embarrassed and uncomfortable when assisting TTGD people”; “Don’t know what name to call the user”; and “Don’t know the user’s personal pronouns, but don’t ask them.”

- “The feeling was what I told you [when seeing a trans user on the service], of embarrassment and surprise too, right?” Red
- “I think it’s called by the male name because that’s what was on the document, something like that. (…) So… her desire was to be a woman.” Golden

#### 4.1.3 Denaturalization of TTGD-identities

This category can be defined by participants not seeing TTGD people as “normal” or “natural” people, even though some participants who had speeches coded in this category reported trying to be respectful to the TTGD users. This category was built from the subcategories “Try to act naturally with TTGD users, despite not considering their identity natural”; “Feel surprised when seeing a TTGD user”; and “Show taboos or prejudices against TTGD people.”

- “Is Cis normal? Hasn’t it changed?” Golden
- “(…) “Am I doing the right thing? Am I really being indifferent?” It’s not indifferent like that. It’s natural, right, to treat naturally, and sometimes it’s difficult, right? We treat it naturally.” Red

#### 4.1.4 Limited pharmaceutical involvement in multidisciplinary teams

This category was developed from the subcategories “Work in multidisciplinary teams” and “Intensity of pharmacist collaboration with other professionals.” The analysis revealed that, although most services included multidisciplinary teams and pharmacists expressed a willingness to engage in collaborative work, they were generally not integrated into these teams. In practice, only one PHC center had the pharmacist actively participating in team activities, including weekly meetings to discuss user cases and service practices. In all other settings, pharmacists worked in isolation, with limited or no involvement in collaborative care planning.

- *“It’s just that we don’t participate much in the meetings of the areas… of the teams, right? We’re kind of left out. That’s right. I don’t know how to answer you.” Gray*

#### 4.1.5 Pharmaceutical services focused on product delivery

This category resulted from the subcategories “Dispensing Medicines (properly or delivering them)”; “Managing administrative processes to access medicines”; and “Incipience of other healthcare initiatives.” This category was created after analyzing the kind of services delivered by the pharmacists to TTGD users, based on their previous experiences. The main practice reported by pharmacists for both cis and TTGD users is dispensing medicines. Some participants reported dispensing medicines without providing guidance, whereas others described dispensing them with due care; however, many highlighted time constraints and structural limitations that hindered the delivery of comprehensive pharmaceutical care. Another practice frequently reported was managing the bureaucratic requirements to access medicines through the Specialized Component of Pharmaceutical Services of SUS. Practices involving deeper pharmaceutical care, such as pharmacotherapeutic follow-up, prescription conferences, and consultations, were incipient. When present, most of these practices were focused on antiretroviral therapy, despite participants’ willingness to provide broader pharmaceutical care.

- “We can’t do the follow-up, but if we can assist, assist the user, for example, doubts, sometimes, teach how their device is for inhalation, insulin, TB [tuberculosis] user, we must try to follow up [the treatment], do you understand? (…) “We spend more time on dispensing because here, only pharmacists can dispense controlled dispensing. (…) It’s not a dispensation, it’s a medication delivery, right? So, this is a medication delivery.” Yellow

#### 4.1.6 Greater contact by pharmacists linked to TTGD outpatient clinic

This category shows that the pharmacists who were linked to the TTGD outpatient clinic had more contact with TTGD users than those who work in other services. This category was created by elucidating how often the pharmacist in each workplace used to see TTGD people, and emerged from “Very often see TTGD people in the service”; “Used to see TTGD people in the service often, but it has reduced”; “Rarely see TTGD people in the service.”

- “Humm…, no. So… As we assist the HIV population and many… a good portion of the HIV population ends up being trans, not just trans, LGBT. So, kind of already… people, already have a care…” Orange
- “And here [currently workplace], there are fewer users compared to the number I worked with there [last workplace, responsible for delivering medicines for ISTs], right? There are many fewer here.” Pink
- “Currently, we have few users who are… identify themselves as transgender. Yeah… when the TTGD outpatient clinic was here within the service, we had more people who attended… this service, right? White

### 4.2 Causal conditions

Causal conditions refer to the factors that have a causal relationship to the central phenomenon (21, 22). The causal conditions that led to the pharmaceutical services and pharmacists’ professional qualifications to provide healthcare for TTGD people were categorized into two categories.

It was identified that the general lack of training and academic experiences in TTGD healthcare, which the participants referred to as insufficient and/or rare, negatively impacted their qualifications to provide healthcare for TTGD users.

#### 4.2.1 Lack of academic experience related to healthcare for TTGD people

This category emerged from the subcategories “Received (or not) training on TTGD-healthcare-related issues” and “Realize the lack of training on TTGD healthcare” (Table 3).

Regarding the pharmacists’ academic experiences, most participants highlighted the absence of training on TTGD people’s healthcare within mandatory undergraduate courses. In their narratives, this lack of academic preparation emerged as a gap that shaped their professional practice. When training was mentioned, it referred to extracurricular activities during undergraduate studies or to more subtle discussions in postgraduate contexts, such as a master’s degree in Public Health or a Multiprofessional Residency in Family Health.

- “Oh, yes, they [the gender identities included in the trans umbrella] can be, it’s… homosexuals, right? Gays, lesbians, right (…) We never had a training, that… that get, said, and talked about trans, what trans is (…) I need training… I would need a training, well… (…) the minimum for us to receive, for… these people and know what problems they face, in terms of health.” Red
- “No [I wasn’t prepared to approach a trans user], because I hadn’t realized yet that this could happen to me, you know? (…) [I had] Zero academic training.” Blue

#### 4.2.2 Lack of workplace training on healthcare for TTGD people

This category was developed based on the subcategories “Isolated experiences of on-the-job training on TTD healthcare” and “no on-the-job training on TTGD healthcare.”

Most participants didn’t have any on-the-job training related to TTGD-healthcare. Some participants had rare on-the-job discussions or formal training; these experiences were superficial (explaining how to approach TTGD people) and exclusive to the professionals who worked in services linked to the TTGD outpatient clinic.

- “What would it be [the gender identities included in the trans umbrella]? (…) I don’t know… (…) I really don’t know [any policy related to trans or LGBTQIAPN+ healthcare]. (…) I don’t think I have ever received any continuing education, right? Of… of assistance to the trans population.” Yellow
- “… at some point or another, this topic [trans healthcare] was… was approached [at work], right? To explain that, how to welcome… Some basic things like this (…) Both since the academic training that I had that… that did not address this in any way. (…) The discussions of the research groups, right? (…) the academic environment in… in graduate school also helped me to have some knowledge about it” Purple
- “As we didn’t have this [training] in a… institutional manner, within our college curriculum, right? If I hadn’t been through these other spaces, I wouldn’t have been prepared… (…) So… I probably wouldn’t know how to ask for their social name, I wouldn’t know… how I should refer to that person because that’s never been taught or said anywhere, right? (…) If I hadn’t had these extracurricular experiences, I would have arrived [at the workplace in my early career] very unprepared because my undergraduate studies didn’t prepare me for this.” White

### 4.3 Contextual conditions

Context refers to specific events or circumstances that locate and explain situations or actions–interactions that make up the causal and interviewing conditions, the phenomenon, and the consequences (21, 22). In this study, the context is described through the conditions from four categories.

#### 4.3.1 Coloniality: the modern colonial CIStem of gender and capitalism

This category was constructed from participants’ discourses, with its theoretical development informed by Maria Lugones’s concept of “Coloniality of Gender.” Lugones’ theory emphasizes how cisheteronormative norms are imposed through social and institutional structures and how power operates through the interlocking systems of race, gender, and capitalism within the modern/colonial world system (26, 27). The word “system” used by Lugones was replaced by the expression “CIStem,” used by the trans-feminist researchers Vergueiro (28) and Nascimento (29), to emphasize the cis/heteronormative matrix that marks social relationships hierarchy in which being cisgender is naturalized while being trans is considered deviant.

The main characteristics of this “CIStem” found in this study are the influences of capitalism and the social cisgender norms on Pharmacists’ Professional and Service qualification. This category was divided into the following subcategories: “General precarious services due to Neoliberal policies and dismantling the healthcare network”; “Cisnormative values as a social standard”; “University’s mandatory subjects run according to CIStem’s norms – excluding TTGD people”; “Healthcare services reproduce discrimination against TTGD people”; and “Exclusion of TTGD People in Cisnormative Networks.”

The influence of the capitalist logic on the services, through the neoliberal policies and the dismantling of primary healthcare settings in the public network of the municipality, was explicit by the general precarious services characterized by the: Lack of workforce; Overload of pharmacists, facing pressure to deal with a big flow of users, which service managers prioritize the delivery of medicines over services related to user care; Poor-quality physical structure in primary healthcare centers, especially regarding the pharmacy structure and professionals, with managers prioritizing a biomedical (medical centralized) structure for the services.

The presence of cisnormative social norms was evident in several situations: some pharmacists did not perceive TTGD people’s identities as “normal” or “natural”; cisnormative approaches were often applied to TTGD users, even by pharmacists who expressed willingness to be “open-minded” and demonstrated some positive attitudes toward TTGD individuals (e.g., orange, golden, blue). Additionally, during the interviews, some pharmacists expressed contradictory positions regarding their willingness to welcome and respect TTGD people. The exclusion of TTGD people in cisnormative networks was evidenced by the limited personal contact that participants (all cisgender pharmacists) reported having with this group. Those with the least contact with TTGD people revealed stronger cisnormative influences on their knowledge and greater difficulty communicating with them. Participants also acknowledged that some healthcare services in the municipality reproduced discriminatory practices against TTGD people.

- “I think [the demand] was for a… for an injectable contraceptive, injectable hormone. I told her “Yes, there is [the medicine in the pharmacy], but for, for, for us to be able to assist, we just need to have a doctor’s prescription, right? So, the ideal is to go through a service and such and if this is prescribed, we can assist.” But (…) I didn’t realize (…) that, in fact, the demand was not for a… for the contraceptive, right? It was for a… for, for a hormone treatment.” Purple

#### 4.3.2 Healthcare regulation structuring specialized services and PHC referral-counter-referral systems

This condition emerged from the subcategories: “Presence of one outpatient clinic specialized in TTGD people’s healthcare in the city public network”; “PHC services are decentralized and responsible for acting as the center of communication between healthcare points, referring users to specialized centralized services when necessary”; and “Communication failures in the health services network.” The other contextual conditions didn’t derive into subcategories.

#### 4.3.3 The poor PNSILGBT implementation in service

The National Policy for Comprehensive Health of Lesbians, Gays, Bisexuals, and Transgender People (PNSILGBT) was established in 2011 to promote inclusive health care, including professional education (30). However, its limited implementation was identified as a contextual condition, based on participants’ reports of not receiving training at work or in the academy regarding LGBTQIAPN+ healthcare and having the TTGD outpatient clinic as the only initiative related to this population in the city.

#### 4.3.4 The changes in the community pharmacist profile/scope of practice

The category is grounded in participants’ accounts and further informed by current legislation and evolving market demands, highlighting the growing importance of pharmaceutical care and collaborative multiprofessional work (17).

Nonetheless, this study found limited multi-professional engagement despite the presence of teams in most services; pharmaceutical activities remained focused primarily on product delivery, with limited provision of pharmaceutical care, even among pharmacists expressing willingness to expand their role. This mismatch contributed to participants’ dissatisfaction with their professional practice.

- “We have no space! There’s no room, there’s no, there’s no structure, [referring to the workspace]. (…) there’s a little room at the front that was the pharmacist’s room… (…) But in the first opportunity, we already lost the room, because there was no space for doctors. (…) I’m very frustrated because we don’t do any work to… to follow anything up. We can’t keep up with ourselves, we can’t do anything, we can only dispense the whole day.” Yellow

### 4.4 Intervening conditions

Intervening conditions are factors that promote or hinder actions or interactions, changing or alleviating the phenomenon experienced (21, 22). The intervening conditions found in this study are described by the following categories.

#### 4.4.1 Social, cultural, and political environments and values

This intervening condition captures the influence of participants’ broader social and political contexts, including personal environments, cultural values, and exposure to political discourse, on their understanding of and attitudes toward TTGD people. Some participants reported internalizing taboos or prejudices rooted in their social circles, which shaped their views and interactions with TTGD individuals. Others described gaining knowledge through political discussions or from friends actively engaged in TTGD healthcare. One participant cited learning from friends who helped establish a specialized outpatient clinic for TTGD people, highlighting how informal social networks can serve as important sources of awareness.

It was found that social, cultural, and political environments and values could influence both positively and negatively the pharmacists’ qualifications to provide healthcare for TTGD people. Some participants’ experiences are examples of this impact. Green had political-environmental experiences and presented more empathy and critical views regarding trans users’ welcoming in health services, when compared to most of the participants. They knew that the TTGD outpatient clinic existed, but didn’t know a lot about its services and medicines provided. However, like most pharmacists, they had difficulty in explaining TTGD-related concepts and had negative experiences communicating with a trans user.

Red identified the presence of prejudices against the LGBTQ+ community in their social environments from a young age and presented cisnormative practices and prejudiced views toward TTGD people. They reported not having contact with LGBTQ+ people or discussions in their personal environment. Red’s discourse reflected a marked lack of knowledge regarding trans-related concepts (such as confusing trans and intersex), specialized services and policies, and management of hormonization, as well as difficulties in communicating with TTGD users.

- “Including contact with trans people, listening to them talk, hearing what they think, what they feel, and what they defend. It’s much more in the political field, right? Then inside academia. I don’t think I’ve ever seen it inside academia.” Green

#### 4.4.2 Occasional experiences related to TTGD people in academic activities and work environments

This intervening condition refers to sporadic or incidental exposure to TTGD-related topics during extracurricular academic activities or within professional settings. Such experiences, though not systematic or in-depth, provided some participants with initial awareness or partial knowledge about TTGD identities and healthcare, influencing their preparedness and attitudes to a limited extent.

Occasional experiences related to TTGD people in extracurricular academic activities and work environments positively influenced the participants’ qualifications to provide healthcare for TTGD people. White and Purple were examples of this influence.

Purple had a few extracurricular academic and work experiences that affected their knowledge; they acknowledged their impact, and we identified it through their answers: they were able to explain TTGD-related concepts, such as gender identities, sexual orientation, and other related notions, and they were familiar with the healthcare services available to TTGD people in the municipality. Purple’s experiences also reduced their difficulty in communicating with TTGD people, as they reported having difficulties in their early career and becoming more comfortable communicating with TTGD users over time. However, they couldn’t describe or explain the main policies related to TTGD people’s healthcare, and didn’t know about the management of hormone use.

- “When I did [cited the name of an extracurricular project that put them in contact with the trans community], yeah… I didn’t, until then I didn’t know much about the difficulties, the… the access barriers, the things that [trans] people had to access health services (…) And then… yeah… I remember people’s speeches, how much it impacted me, knowing the prejudices they suffered in the service.” White

#### 4.4.3 Relate with narratives of TTGD people

This intervening condition refers to participants’ previous contact with TTGD individuals across various contexts, which influenced their perceptions, attitudes, and practices. These narratives emerged from experiences in three main domains: involvement in social movements; personal environments, such as friendships or family relationships; and professional experiences, including assisting TTGD users in healthcare settings or working alongside trans colleagues. The depth and nature of these interactions shaped how participants engaged with gender-diverse individuals in their professional roles.

The contact with TTGD people’s narratives had positive influences on pharmacists’ qualifications to provide healthcare for TTGD people, reducing the lack of knowledge and the difficulty in communicating with TTGD users. Blue, Pink, Orange, and White experiences exemplify the impact of these intervening factors.

Informal and experiential learning with trans narratives emerged as a key learning pathway for participants, often through TTGD peer educators, colleagues, or direct contact with users in services. These interactions contributed to greater awareness of TTGD health needs and approaches to communication and bonding with this population. For example, Blue and Pink highlighted learning opportunities with trans women who were involved in HIV prevention projects, while Orange and Blue emphasized that daily work in services linked to the TTGD outpatient clinic provided contact that fostered greater respect for diversity. Nonetheless, these learning experiences were insufficient for participants to feel prepared to provide counseling on hormone use, and they revealed persistent difficulties explaining TTGD-related concepts, distinguishing gender identity from sexual orientation, and recognizing national policies or structural aspects of TTGD care. Some discourses also reproduced cisnormative perspectives, despite attempts to show respect for TTGD users.

White’s trajectory included several intervening conditions that supported their qualifications to provide care for TTGD people, encompassing personal, academic, and professional environments, as well as contact with TTGD narratives. Friends involved in mobilizing the creation of the local TTGD outpatient clinic, extracurricular activities with social movements and NGOs, and work experiences all contributed to strengthening values such as respect for diversity and expanding their knowledge. As a result, White demonstrated an understanding of TTGD-related concepts, identified specialized services as well as national and local policies, and reported no difficulties in communicating with TTGD users. Nonetheless, gaps remained, as White incorrectly stated that the outpatient clinic did not dispense hormones and acknowledged not feeling prepared to provide counseling on harmonization.

- “Yes… we had no training regarding pharmacological treatment. But we go after it, right? Yeah… I, through a research project with Fiocruz, and I work directly with a peer educator, who is a trans woman (…) who is part of the team. In which I constantly learn from her, right, that she seeks to do this fieldwork for us to map, right…” Blue
- “So, it was in college, and I had a classmate who… She was a trans woman. (…) And to this day, she works with… this type of LGBT cause, with HIV issues, with trans issues. So, I had a certain amount of knowledge, even because of her, right?” Pink
- “So sometimes it’s hard to get into people’s heads, but one thing I’ve learned here [at their current workplace, linked to the TTGD outpatient clinic] is that kind of… Open your head more, change… like, ok, but that’s a person who she is… who she is, and that’s the most important thing, right?” Orange

### 4.5 Action/interaction/strategies

In grounded theory, actions/interactions/strategies refer to the responses individuals develop in relation to the conditions that constitute the phenomenon under study. These actions should address the questions: “What meaning (in the form of a problem, goal, etc.) was given to these conditions or set of events? What particular action–interaction was taken to manage the problem or reach that goal?” (21, 22).

Within this analytical framework, participants in this study employed different strategies to address both their own and the service’s qualifications when providing healthcare for TTGD people. Some developed respectful interactions, such as using social names, correcting colleagues, and offering training, others did not develop such strategies and instead adopted avoidance practices that reflected their lack of qualification, leading to harmful behaviors like neglecting social names or absence of deeper care. Positive intervening conditions favored more respectful and helpful strategies, whereas negative conditions led to the opposite. Some strategies operated unconsciously, including cisnormative guidance regarding medicine access and use. These actions and interactions were analytically organized into six categories, two with subcategories and four standalone categories.

#### 4.5.1 Actions regarding the use of the user’s social name and personal pronoun

This category refers to the strategies adopted by participants when addressing TTGD users in relation to their social name and preferred pronouns. It was constructed from the subcategories: “Asking for social name and personal pronouns when in doubt” and “Calling the user by the wrong (non-preferred) name.” These actions reflect varying levels of preparedness and sensitivity among participants. While some actively asked users how they would like to be addressed, demonstrating openness and a willingness to respect identity, others admitted to not using the social names, sometimes recognizing their mistake and expressing discomfort or regret.

- “I think it’s a female name. And… who looked like a man… (…) And I thought he was opening the process [to access medicines] for someone else, but it was him. (…) But then… I didn’t ask, but… then I felt it was for him, you know? I didn’t ask.” Red

#### 4.5.2 Interactions with peers toward adverse situations related to TTGD people’s healthcare

This category encompasses strategies involving peer-to-peer interactions when facing difficulties or uncertainties related to the healthcare of TTGD users. It includes actions such as seeking guidance from colleagues perceived as more knowledgeable, offering training or information to coworkers (even if informally or as isolated initiatives), and addressing or correcting biased behaviors observed in the workplace. These strategies reflect both individual efforts to improve care and attempts to influence the broader professional environment, though they often occurred without institutional support or systematic training.

- “Then, when I had any questions, I asked *[colleague name]*, right? She was a pharmacist there…” Pink
- “And the team there was not prepared to assist, so they kept making some types of jokes. Some things like that, after the user left. Not at the time of service. And then, I remember that I went to talk to the team, and then I did training for the team.” White

#### 4.5.3 Providing fast and superficial care services to all users

Pharmacists reported providing fast and superficial care to users as a strategy to cope with the services’ focus on dispensing medications and the limited opportunities to offer comprehensive pharmaceutical care. This category reflects an approach of delivering brief, task-oriented care to all users, regardless of gender identity. However, these constraints particularly hindered interactions with TTGD users, with whom pharmacists often experienced communication difficulties. Participants emphasized efficiency, frequently measured by the number of users served. The prioritization of volume over individualized attention underscores systemic pressures and institutional norms that may unintentionally perpetuate inequities in healthcare delivery.

- “The patient comes just to collect the medicines.” Pink
- “We don’t have time *[to attend the multiprofessional team meetings]* because it’s controlled medicines dispensation all the time. (…) It’s not a dispensation, it’s a medication delivery, right? (…) We rarely do some [deeper care] work.” Yellow

#### 4.5.4 Avoiding deeper care/discussion with TTGD users

This category refers to the strategy adopted by some participants of limiting their interactions with TTGD users to basic or superficial care, deliberately avoiding more in-depth pharmaceutical care, even in services where such care was feasible and routinely provided to cisgender users. This distancing may reflect discomfort, lack of knowledge, or fear of saying something inappropriate. As a result, opportunities to provide more comprehensive, person-centered care were often missed, reinforcing barriers to equitable healthcare access for TTGD people.

- “I don’t even know how to talk to something I don’t know, okay? It’s a completely unknown universe. (…) I didn’t know what to say [referring to talking to a trans user] … he, she… (…) I should have written it [the questions on how to manage the situation], I should have remembered, right? To have spoken to colleagues, right. But I didn’t.” Red
- “And since we also don’t approach it or ask or not… huh? Suddenly, the person doesn’t even feel welcomed, right?” Green

#### 4.5.5 Guiding pharmaceutical services in a cisnormative way

This category describes the practice of pharmacists guiding medicine use based on cisnormative assumptions, particularly regarding prescriptions and contraindications related to pregnancy. This approach often overlooks or inadequately addresses the specific needs of TTGD users by applying standard protocols designed for cisgender patients.

- “When you’re opening the process [to access medicines with contraindications for pregnancy], the user also needs to bring the notification, right? The prescription with the post-information form, which says whether they use contraceptives, right? They have to bring the negative beta HCG test. (…) So… they will bring the… term post-information, which has two types. (…) There is one for women over 55 years old. So what? It is no longer considered a fertile age, and it is also for men. (…) And there is also a term for women up to 55 years old.” Golden.

#### 4.5.6 Referring TTGD users to other services

This category describes the common practice of pharmacists referring TTGD users who seek care at the pharmacy to transgender outpatient clinics or other specialized health services, primarily those related to STIs. In participants’ accounts, this often appeared as an automatic referral to the TTGD outpatient clinic, regardless of whether the demand was specifically related to gender-affirming procedures. Even when the request could have been managed within primary healthcare, pharmacists tended to refer TTGD users instead of addressing the need in their own service. This strategy reflects both pharmacists’ perceived lack of expertise or resources and the institutional tendency to centralize TTGD healthcare in specialized settings. While intended to ensure appropriate care, frequent referrals contribute to fragmented care and limit opportunities to build trust within the initial healthcare environment.

- “Sometimes, we receive some questions, the issue of… hormones, right? The medicines that SUS provides for the transition. Sometimes, some people wonder, but since it’s not here with us, I don’t have that much access. We only refer them [to the TTGD outpatient clinic] …” Pink
- “I think [that the TTGD outpatient clinic] ends up absorbing everything like that because… Because basically, it [the TTGD outpatient clinic] was created not only with the intention of transitions, but of… of assistance, of trans [people’s] demand itself. (…) Primary care [confirming].” Orange

### 4.6 Consequences

The consequences are outcomes of actions and interactions (21, 22). In this study, the consequences were described by seven categories. The first emerged from subcategories, while the others didn’t develop into subcategories.

#### 4.6.1 (Un)preparedness feelings

The first category was characterized by participants’ reports of preparedness feelings when providing care for TTGD people and was divided into the subcategories “Feel unprepared to provide care related to hormone use”; “Feel unprepared to provide general care for TTGD people”; and “Feel prepared to provide care for TTGD people.”

It was possible to observe that the pharmacists felt unprepared to provide general care for TTGD people, especially to provide care related to hormone use, and only one of the participants (White) felt prepared to provide care for TTGD people. Notably, White attributes their preparedness to the experiences, conceived as intervening conditions, encountered during the qualification process. Although experiencing these conditions, they didn’t feel prepared to provide care related to hormone use for TTGD users.

- “I had a feeling like this. You know when you feel… Without any knowledge about a subject. And then you, you wanted to have more knowledge to talk more, to deal with this situation more.” Golden
- “So, I think that if we have, you know… these medicines in the SUS, anyway, it will be necessary to have a broad discussion on the clinical part [of hormone use], right? (…) So that we are aware of what we are doing, so that we know how to identify possible problems related to this “hormone therapy,” right? (…) as it is not something we do in our daily lives, it is not knowledge that we have, easily accessible in our heads. (…)” White

#### 4.6.2 Negative feelings when making mistakes related to user gender identity

This category was characterized by the participants’ reports of feeling bad when calling a TTGD user by the wrong (not the preferred) name or pronoun.

- “I had already identified [the user] as… as being a male person. And then, in the middle of the conversation, she said, no, but ah… I’m a woman… It’s her, it’s not him (…) I was totally embarrassed.” Purple
- “I was not given her social name. And since it was in the waiting room, I called by the civil name, which was what was on my agenda, which caused a very big embarrassment for the person, right? (…) it was a very hard job for me to demystify that first assistance, so… and… and… win, right, the person’s trust, empathy…” Blue

#### 4.6.3 Feelings of being distant from the users/not having a link with them

This consequence category was built from participants’ reports of feeling distant from the users when delivering pharmaceutical services to them, relating it to users’ fears of being mistreated or not welcomed, and with pharmacists’ lack of contact with the TTGD community.

- “We realize that the person, she, she herself already puts herself in a position of… (…) Afraid she might not be treated properly.” Blue

#### 4.6.4 Perception of the need for more training to provide care for TTGD people and willingness to learn more

This category was characterized by participants’ recognition of their lack of training and their need to learn more about TTGD people’s healthcare, particularly regarding hormone management, with most participants expressing interest in further learning on the topic.

- “I think I’ve come a long way from what I was when I recently graduated. (…) But I know I still have a lot to learn. And I recognize that I need to improve and have a lot more knowledge and… I’m certainly interested [in TTGD healthcare training].” Purple

#### 4.6.5 Realize the need to insert TTGD health issues in pharmacy curricula

This category was constructed from participants’ accounts, emphasizing the need to formally incorporate TTGD health into undergraduate pharmacy curricula as mandatory content. Participants described gaps in their academic training regarding TTGD health, which resulted in unpreparedness to address TTGD health needs within routine pharmaceutical practice. The inclusion of TTGD-related topics was framed as essential to ensuring the universal provision of pharmaceutical care, in line with the principles of equity and comprehensiveness within the SUS.

- “Health professionals need to have training on this topic, so they need this to be included in the university curricula. That they leave [university] already prepared to deal with the health issues of the trans population.” White
- “I think it [including TTGD healthcare in pharmacy curriculum] is fundamental because it is precisely during the undergraduate studies that we need to experience and access the basic content so that in our professional life, we… go deeper and refine and improve, right? Developing, but I consider it as content that is, huh… essential, that should be mandatory, right?” Green

#### 4.6.6 Superficial pharmaceutical healthcare for TTGD people

This consequence was identified through analysis of the types of services provided by pharmacists to TTGD users. These services were primarily characterized by medication dispensing, referral to other services, delivery of medical prescriptions to users, counseling related to medicines or services, and management of administrative processes. Most services were found to be superficial, with dispensing activities rarely involving pharmaceutical care and largely limited to the delivery of medicines. More comprehensive care was observed mainly within HIV specialized healthcare facilities.

- “We even receive a lot like this at the pharmacy, we have the dispensation of Spironolactone, right? And some medications (…) I notice because there are a lot of people who are transitioning. And they are usually followed up elsewhere. (…) No [we don’t follow them up], because the contact is very quick. It’s just dispensation (…) it was very fast.” Yellow
- “There are a lot [of dispensing for trans people] of controlled medications. Yes… for trans woman, yes… we see that there are lots of contraceptives, right, that take it.” Gray

#### 4.6.7 Centralization of TTGD people’s healthcare in specialized services

This consequence was identified based on pharmacists’ reports indicating that the healthcare of TTGD people is predominantly delivered in specialized TTGD outpatient services, which are sought for issues spanning primary, secondary, and tertiary levels of care. Pharmacists working in other healthcare settings reported limited contact with TTGD users within their own services, often interacting with them only before referral care had been transferred to another facility. In contrast, pharmacists working in services linked to specialized TTGD outpatient clinics described more direct and continuous contact with TTGD users.

- “They [TTGD users from all regions of the city] end up coming to the [TTGD outpatient clinic, linked to the pharmacy] now (…) I imagine, but it is more accessible, they [the professionals] are more open people.” Orange
- “I believe so [trans people end up preferring to go to the TTGD outpatient clinic that they work at over other health centers], because here they feel more welcomed, because it is a TTGD outpatient clinic. So, the service is more targeted, right? It reduces inequalities a little…?” Blue

## 5 Discussion

The findings of this study reveal essential gaps in the qualifications of pharmaceutical services to provide care for TTGD people, exposing structural and sociopolitical challenges within the Brazilian health system. Consistent with prior international research on health professionals in TTGD health, the results indicate that gaps in academic curricula and professional practice contribute to difficulties in communication, superficial consultations, and a reliance on referrals to specialized services (31–35).

These qualification gaps not only compromise professional performance but also manifest in the emotional experiences of pharmacists. Analytically, feelings such as unpreparedness, discomfort when making mistakes, and emotional distancing emerged as consequences of their actions, interactions, or strategies to provide care for TTGD people.

The phenomenon we are analyzing is broad and complex: the concept of qualification extends beyond technical training, encompassing the knowledge and skills acquired through education and socialization, and is defined as the potential to perform tasks in the workplace (2, 3, 6). It is not a static attribute, but rather a multidimensional and socially constructed process shaped by the dynamics of capital and labor, linking education and work and reflecting broader power relations (4, 5). While clinical and cultural competences address historical gaps, they remain insufficient to overcome broader structural and organizational barriers, which continue to limit the effectiveness of healthcare services (36). The limited involvement of pharmacists in multiprofessional teams, the predominance of product-centered activities, the structure of healthcare regulation, and the lack of managerial investment in pharmaceutical careers are examples of broader barriers, as also reported in previous studies (37–39).

We found that Primary Health Care (PHC) pharmacists were frequently unable to provide continuous support to meet the needs of TTGD individuals. Gaps in professional qualifications directly compromise the effectiveness of health services. Previous research indicates that TTGD people often avoid or are excluded from SUS PHC, while pharmacists can serve as key, and sometimes sole, points of contact (16). Although the role of pharmacists in trans health is increasingly recognized (12), most participants in this study reported feeling unprepared to provide TTGD care, highlighting the need for targeted training, the decentralization of LGBTQIA+ inclusive services, and full implementation of the National Policy for LGBTQIA+ Health. The centralization of care in specialized services replicates historical patterns observed in HIV/AIDS policies, which, despite promising universality, integrality, and equity, were concentrated primarily in specialized clinics and emergency services (40).

Current evidence highlights that the qualifications of healthcare professionals directly shape health outcomes for TTGD users (31, 41–44). In Brazil, following guidelines from the Pan American Health Organization, the Brazilian Ministry of Health, and the Federal Council of Pharmacy, pharmacists are expected to expand their role in primary care by supporting access to medicines and contributing to integrated, comprehensive care. Resolutive PHC has been associated with improved health indicators, enhanced clinical effectiveness, greater user satisfaction, and reduced inequities (45). Recent institutional and regulatory measures have reinforced pharmacists’ inclusion in PHC teams, although this process is still being consolidated (38).

In the context of trans health, studies emphasize the expanding role of pharmacists in providing respectful and unbiased care, which includes pharmacotherapy management, laboratory monitoring, counseling, hormonization support, and preventive services (11, 12). In Brazil, trans users point to the urgent need for professional training on gender diversity, welcoming practices, and the consolidation of accessible, inclusive, and comprehensive models of care to overcome fragmentation and respond to their complex health needs (46).

From the perspective of professional identity formation, the findings can be interpreted by considering how education and the practice environment function as central contexts for professional socialization and the development of professionalism. According to this framework, formal education introduces foundational knowledge, values, and expectations of professional conduct, while workplace settings reinforce and shape these elements through experiential learning, role modeling, and organizational culture (47). When issues related to gender diversity are insufficiently addressed across both educational and practice settings, gaps in their qualifications may persist, influencing pharmacists’ interactions with TTGD populations.

Within the framework of SUS regulations, workforce development, and service organization, the qualifications of healthcare professionals for TTGD care must encompass technical, political, and cultural dimensions. The expansion of social participation and health democratization in Brazil has fostered inclusive policies for vulnerable groups, including LGBT populations, as strategies to reduce inequities and guarantee rights (9). Milestones such as the Brazil without Homophobia program (2004), LGBT representation in the National Health Council (2006), the SUS Transsexualizing Process (PTr, 2008; 2013), and the National Policy for Comprehensive LGBT Healthcare (PNSILGBT, 2011) illustrate the institutionalization of this agenda, as these policies were created to address historical inequities, promote integrality, and meet the specific needs of LGTBQIAPN+ populations (9, 10).

The PTr focused primarily on body modification services for trans people and has been widely criticized for its pathologizing, cisnormative, and binary approach. In contrast, the PNSILGBT adopted a broader approach, aiming to eliminate prejudice and reduce inequalities in health care for the LGBT population by ensuring comprehensive care. The policy establishes that SUS facilities should implement welcoming practices, respect and use social names, and provide hormonization services and gender-affirming surgeries. In addition, it mandates the inclusion of LGBT health topics in both academic curricula and workplace training for SUS workers and healthcare students. However, the lack of PNSILGBT implementation has been consistently documented as a persistent challenge in providing healthcare for the LGBTQIAPN+ population in Brazil (48, 49). Nevertheless, both initiatives represent important advances in the struggle for trans rights in Brazil (9, 10, 50).

Despite progress, our findings highlight that TTGD care within SUS remains fragmented, underfunded, and highly centralized, with specialized clinics only minimally integrated into broader health services (9, 50, 51). Importantly, even pharmacists working in these centralized specialized settings are often unprepared and continue to occupy peripheral roles within interprofessional care teams, limiting their ability to contribute to comprehensive and continuous care.

While legislative frameworks and professional guidelines support an expanded pharmacist role, without infrastructure and proper qualifications, these legal advances are underutilized (37, 38, 52). Poor-quality PHC facilities, combined with biomedical and physician-centered managerial priorities, reflect Foucault’s (53) dynamics of medical power and biopolitics, perpetuating professional marginalization and limiting comprehensive care for TTGD people (52).

Tomim (52) and Agostini (45) highlight a tension in SUS PHC between a territorialized, interprofessional model based on comprehensive care and a neoliberal managerial logic focused on targets, productivity, and the financialization of public policy, which undermines service quality and fosters a form of necropolitics that marginalizes socially vulnerable groups, weakening global health leadership. The results found in our study underscore the negative impact of neoliberal policies on the qualifications of professionals and health services, resulting in pharmaceutical practices that are superficial, centralized, medication-focused, and that marginalize TTGD people in primary care.

Beyond structural and policy-related factors, broader sociocultural patterns also shape the qualifications of pharmacists and the services they provide. This study revealed several manifestations of coloniality, underscoring the need for health sciences to engage in dialogue with social sciences. Such colonial phenomena can be understood through Lugones’s concept of “Coloniality of Gender” (26), developed in critical dialogue with Aníbal Quijano’s concept of “Coloniality of Power,” which describes the global Eurocentric capitalist system of domination established through colonialism. While acknowledging the relevance of Quijano’s framework, Lugones critiques it for overlooking gender and sexuality as constitutive dimensions of colonial domination. By centering the imposition of a cisheteronormative and patriarchal gender system, Lugones advances by expanding the understanding of how power operates through the interlocking structures of race, gender, and capitalism within the modern/colonial world system (26, 27). While Quijano emphasized how colonialism relied on the social classification of populations through the invention and imposition of the idea of race, generating power relations of superiority and inferiority between colonizers and colonized peoples (27), Lugones goes further by exploring, from a decolonial feminist perspective, how European colonialism imposed gender systems on colonized peoples, producing binary oppositions and hierarchical social categories related to gender and sexuality (26).

These historical and structural dimensions of coloniality provide a lens through which to interpret the limited engagement of pharmacists with the TTGD community in SUS, linking broader sociocultural patterns to observed professional practices. The limited contact between most pharmacists (all cisgender) and the TTGD community reflects these enduring structures, illustrating how broader patterns of coloniality continue to influence healthcare access and professional practices (27, 28).

We also found that professionals who actively engaged with TTGD narratives, through academic or professional experiences, developed stronger qualifications and greater sensitivity. Previous evidence discusses that although healthcare professionals are not solely responsible for systemic shortcomings, they retain agency in shaping their own trajectorie (49). In contrast, Paulino (54) conceptualizes the “discourse of not-knowing,” in which professionals attribute gaps in knowledge to structural failings while simultaneously concealing their own irresponsibility. This strategy illustrates how appeals to “lack of knowledge” can be used to obscure institutional failures in implementing inclusive policies.

Within this context, qualifying pharmaceutical services for TTGD care goes beyond individual competencies, as it is a socially mediated process shaped by education, work organization, and health policies. Advancing inclusive care, therefore, requires not only technical expertise but also critical engagement with structural, relational, ethical, and systemic dimensions that shape service provision and the recognition of TTGD populations as subjects of rights (36). This leads to a shift to the broader concept of qualifications, which integrates individual competencies with the social, cultural, political, and organizational conditions to enable services to meet the needs of Trans, Travesti, and Gender-Diverse populations, particularly through the involvement of TTGD professionals and users.

The pharmacists attributed gaps in their academic background regarding TTGD care to limitations in their ability to provide comprehensive healthcare. Previous evidence highlights that the absence of TTGD health topics in curricula and professional practice contributes to communication challenges, superficial consultations, and excessive reliance on referrals to specialized services (33, 34, 55). Ferreira and Nascimento (9) support our findings, noting that professional training is closely connected to academic education and emphasizing the need to integrate gender and sexuality topics into undergraduate curricula. While this study showed that extracurricular undergraduate experiences can yield positive outcomes, their limited and optional nature restricts access, creating disparities in knowledge and practice. To address this, trans-related content should be integrated into mandatory curricula, experiential learning, and interprofessional programs (15, 56).

The literature underscores that training focused solely on trans identities, technical knowledge, or political awareness is insufficient to overcome barriers that TTGD people face across health services. Technical-only approaches tend to universalize care, reinforcing binary and heteronormative norms, and shaping practices as corrective and disciplinary, excluding non-conforming users. While awareness of rights and anti-discrimination measures is necessary, effective trans-inclusive care requires formative processes grounded in lived experiences, combining ethical-political orientations with technical skills to ensure respectful, non-discriminatory access, recognition of social names, and responsiveness to diverse health needs, including body modification (50).

This study exposed that opportunities to learn about TTGD healthcare in the academic pathway were optional and self-initiated rather than systematically embedded in training, indicating a gap in strategies for qualifying pharmacists to address those health needs. Additionally, engagement with human rights discourses fostered greater awareness, but a decisive factor was exposure to TTGD narratives. Encounters during academic training or professional practices with trans peers challenged cisnormative assumptions. Pharmacists with sustained contact reported more profound changes in skills and attitudes, suggesting that these narratives were turning points in reshaping care. Extension activities and the increasing presence of TTGD individuals in universities have proven transformative, reshaping professional education and approaches to care. Nevertheless, previous evidence emphasizes that training and continuing education strategies are essential for fostering reflection on professional practices, supporting change, and preparing leaders, managers, and health professionals to effectively address TTGD health needs (9).

The lack of TTGD colleagues formally employed in health services highlights the need to strengthen the presence of TTGD professionals in healthcare teams. Evidence suggests that the inclusion of TTGD professionals, particularly peer navigators, in health initiatives not only broadens approaches but also counters the delegitimization of TTGD identities, promoting more inclusive training and professional practice, and enhancing users’ trust in health services. Involving LGBTQIAPN+ individuals as co-trainers further strengthens outcomes by incorporating lived experiences and enhancing professional attitudes (57–60).

In this sense, exposure to diversity, whether through direct professional interaction or structured training, emerges as a key factor in shaping healthcare practices. Our findings resonate with studies demonstrating the positive impact of inclusive health content during professional education (30, 61). However, the contradictory positions observed in some pharmacists’ narratives raise questions about whether their statements reflect a genuine commitment to inclusive care for TTGD people or an attempt to align with socially expected, “politically correct” discourse. This ambivalence suggests that professional discourses are shaped not only by personal beliefs but also by broader social pressures, reflecting tensions between normative expectations, cultural backgrounds, and actual practices in healthcare delivery (62).

Rocon (42) argues that encounters with trans signs generate a productive discomfort that renders issues such as social names and discrimination visible in daily practice, compelling cis health workers to reconfigure how they listen, work, and relate, transformations that cannot emerge from the mere transmission of concepts. Learning from trans experiences thus constitutes an etopoietic process, enabling a reconfiguration of subjectivity that challenges binary and heteronormative frameworks. Respectful and affirming interactions, such as the consistent use of social names and pronouns, are essential to fostering dignity and trust. Equally important are individualized and comprehensive approaches that address specific needs, along with communication, recognition of vulnerability, and practice grounded in professional knowledge (63).

Cultural competence, defined as the integration of knowledge, skills, attitudes, and behaviors to enhance cross-cultural communication and relationships (64, 65), has been applied to guide interactions and improve professionals’ knowledge and attitudes. LGBTQ+ cultural competency training effectively improves knowledge, skills, and attitudes, primarily with an interdisciplinary and multimodal approach (36). Communication abilities emerged in the interviews as a crucial dimension for delivering humanized and horizontal care, recognizing users’ trajectories and knowledge, and enabling professionals to interact beyond their own social bubbles.

This study’s qualitative design provides in-depth, contextualized insights into pharmacists’ experiences in primary and secondary care units of the SUS, in southern Brazil; therefore, findings may not be generalizable to other regions or settings. Researchers’ prior experience and professional background may have influenced data interpretation, and translation from Portuguese to English may have led to minor nuances being lost. Nonetheless, theoretical saturation, professional translation and review, as well as the validation procedures, support the credibility and transferability of the results. The study is also limited by the exclusive participation of cis individuals among both participants and researchers. Although TTGD students were invited to join the team, none participated in conducting the investigation, possibly due to the limited number able to access and remain in academic settings given cisnormative barriers, as well as restrictions related to project funding for scholarships. No TTGD pharmacists were identified during recruitment, reflecting their underrepresentation in the healthcare workforce and suggesting the hypothesis that their experiences may differ from those captured in this study. These factors highlight the need for future research on the qualifications of pharmaceutical services from the perspectives of both TTGD professionals and users.

## 6 Conclusion

This study highlights the need for changes in the qualification of healthcare services and professionals, including service structure, workforce allocation, and pharmacists’ roles, promoting interprofessional collaboration and integration across the public health network. Engagement with TTGD narratives, enhanced academic and professional training, and the implementation of the PNSILGBT are crucial to combat stigma, address knowledge gaps, and ensure effective care. The lack of training, alongside cisheteronormative, capitalist, and neoliberal contexts, contributes to prejudice, service precariousness, and the centralization of care in specialized settings.

Future research should explore interventions integrating TTGD care into professional training and models that actively include pharmacists in multidisciplinary teams. Emphasis should be placed on approaches fostering engagement with TTGD narratives, as these experiences are particularly effective in transforming professional perspectives. Studies assessing how policy implementation and institutional support influence professional qualifications are also warranted. Comparative research across regions and health systems may further expose structural barriers and strategies for strengthening inclusive care for TTGD populations.

Advancing inclusive pharmaceutical care requires a decolonial and multidimensional approach beyond technical competence, integrating clinical, cultural, ethical, relational, and systemic skills with structural and organizational transformations. Inclusive curricula, continuing education, and interprofessional collaboration are essential to overcome structural barriers and cisnormativity. Policies such as PNSILGBT provide a framework, but their impact depends on professional training, managerial support, and network integration. Ultimately, equitable, respectful, and comprehensive care, particularly in PHC, relies on humanized approaches, centering TTGD voices and narratives, and recognizing pharmacists as transformative agents capable of driving innovative, sustainable, and rights-oriented changes within the SUS.

## Data Availability

The original contributions presented in this study are included in this article/Supplementary material, further inquiries can be directed to the corresponding author.
